# Teaching the technical performance of bronchoscopy to residents in a step-wise simulated approach: factors supporting learning and impacts on clinical work – a qualitative analysis

**DOI:** 10.1186/s12909-021-03027-6

**Published:** 2021-12-02

**Authors:** Anne Kathrin Eickelmann, Noemi Jelena Waldner, Sören Huwendiek

**Affiliations:** 1 Department of Anaesthesiology, Intensive Care Medicine, Emergency Medicine, Transfusion Medicine, and Pain Therapy, Protestant Hospital of the Bethel Foundation, University Hospital OWL, University Bielefeld, Bielefeld, Germany; 2 Institute for Medical Education, Department for Assessment and Evaluation, University of Bern, Bern, Switzerland

**Keywords:** Bronchoscopy training, Cognitive load theory, Simulation

## Abstract

**Background:**

The ability to perform a bronchoscopy is a valuable clinical skill for many medical specialities. Learning this skill is demanding for residents, due to the high cognitive load. Lessons learned from cognitive load theory might provide a way to facilitate this learning. The aim of this study was to investigate residents’ perception of factors that support and hinder learning, as well as outcome and acceptance of a workshop on flexible bronchoscopy.

**Methods:**

Three half-day workshops were designed to teach 12 residents the basics of handling a flexible bronchoscope. They consisted of four phases that alternated between short theoretical aspects and longer practical situations. The practical phases focussed initially on manoeuvring a bronchoscope through holes in panels inside a box, and then on examination and practice using a three-dimensional printed model of the bronchial tree. Afterwards, three audio- and video-recorded focus groups were conducted, transcribed and coded, and underwent reflexive thematic analysis.

**Results:**

Analysis of the focus groups defined two themes: (1) factors that supported a safe and positive learning environment were optimised for intrinsic load, simulated setting, absence of pressure, dyad practice (working in pairs), small group sizes and playful learning; and (2) impacts on clinical work were perceived as high levels of learning and improved patient safety. The residents did not report factors that hindered their learning. Some suggestions were made to improve the set-up of the wooden box.

**Conclusions:**

The half-day workshop was designed according to several factors, including cognitive load theory in a simulated setting, and creation of a safe and positive learning environment. The residents perceived this as supporting learning and patient safety. Further studies can be designed to confirm these results in a quantitative setting.

**Trial registration:**

This study was not interventional, therefore was not registered.

## Background

Learning complex procedures that require psycho-motor skills can be very demanding for hospital residents [1]. During bronchoscopy training on the Intensive Care Unit, inexperienced young residents can become overwhelmed with the learning they need to follow directly on patients. Sewell et al. reported similar problems while investigating cognitive load during colonoscopy training [1]. They stated that, ʻa less experienced learner and a more complex procedure would both increase the likelihood that the supervisor will need to take overʼ.

In both of these situations of the traditional apprenticeship model (i.e., learning in the workplace from a more experienced colleague) the learners can suffer from cognitive overload [2]. This state was described by Sweller in 1988 with the first report on cognitive load theory (CLT) [3]. CLT is based on the concept that learning requires interactions between working memory and long-term memory, where the capacity of working memory is limited, unlike that of long-term memory [4, 5]. If the capacity of the working memory is overloaded, learning is hindered [6]. This might be due to learner inexperience, or to the complexity of the subject (i.e., the intrinsic load) or its presentation (i.e., the extraneous load). The intrinsic cognitive load can be increased by the complexity of a psycho-motor skill to be performed, such as bronchoscopy, or by the learner’s lack of knowledge (e.g., of handling a bronchoscope, of the anatomy of the airway, of important landmarks). Factors that lead to an increase in the extraneous load in the bronchoscopy setting include the following: patient factors (e.g., movement, coughing, deteriorating oxygen saturation); noise (e.g., alarm tones, people talking); and distractions (e.g., colleagues asking questions). To counteract this, recommendations have been derived from CLT that aim to decrease the intrinsic and/or extraneous loads [7–9]. For example, a lower intrinsic load can be achieved by reducing the complexity of the skill being learned, while the extraneous load can be reduced by avoiding distracting the learner’s attention during the procedure.

The literature on flexible bronchoscopy training was summarised in three reviews, in 2013 and 2017 [10–12]. These showed that practice using a simulated approach provides the learner with better training than the traditional apprenticeship model. Since then, new technologies have opened up further possibilities for teaching, such as three-dimensional (3D) printing and virtual reality [13, 14].

However, there remains a lack of literature that combines various teaching approaches with CLT findings, and that examines this from the learner’s perspective. This project thus aimed to investigate how residents perceive the learning of bronchoscopy in a step-wise simulation that follows the lessons learned from CLT. The focus was on the factors that support and hinder this learning, and on the outcome and acceptance of the project. The study might also help to understand better how to set up feasible training that enhances learning, while reducing the time and personnel requirements compared to the traditional apprenticeship model.

## Methods

### Participants

The participants in this study were residents of the Department for Anaesthesiology, Intensive Care Medicine, Emergency Medicine, Transfusion Medicine, and Pain Therapy of the Protestant Hospital of the Bethel Foundation. Participation in the workshop was offered to all of the residents at the hospital who had little or no experience in bronchoscopy. A total of 12 residents volunteered to take part in the workshop, which included one resident of the Department for Diagnostic and Interventional Radiology and Paediatric Radiology who asked to participate through contacts with the other residents. The workshops took place on three separate occasions in February 2020, with four participants involved each time.

### Workshop

The goal of the half-day workshop was to teach the residents the basics of bronchoscopy. To achieve this, a high proportion of practical work was used, which was accompanied by theoretical input. The theoretical parts were reduced to the extent that only the aspects necessary for the basic understanding were included. Table 1 gives the details of each part of the workshop.

**Table 1:** Content, duration and objectives of each part of the half-day workshop

Part	Aspect	Content	Duration (min)	Objectives: After this part of the workshop, the residents will…
#1	Theoretical	- Reflection on the use of bronchoscopy in anaesthesia (group meeting with flipchart)	30 min	- have refreshed their knowledge about the indications for bronchoscopy in anaesthesia
		-Introduction to bronchoscopes (PowerPoint® presentation and examination of various bronchoscopes)		- be able to name the components of the bronchoscope and explain their function
				- be able to prepare a bronchoscope for use on the patient
#2	Practical	Manoeuvring two different bronchoscopes through holes in a wooden box	60 min	- be able to coordinate the movements of their hands with the manipulation of the tip of the bronchoscope, to manoeuvre the bronchoscope through the holes of the wooden boards
Break		Refreshments	30–45 min	
#3	Theoretical	Introduction to human airway anatomy (PowerPoint® presentation)	30 min	- have refreshed their knowledge of the bronchial tree and its parts
#4	Practical	Manoeuvring two different bronchoscopes through a three-dimensional printed airway	60 min	- have learned to move the bronchoscope through a human airway
				- have started to practice a structured bronchoscopy, with inspection of the different parts of the bronchial tree, one after the other
				- think of bronchoscopy as a valuable skill

To practice bronchoscopy of the human airway, many different commercial manikins are available. These are intended to be close to reality, and can be used effectively for training purposes [15, 16]. A disadvantage is the high price, which often means that only a single simulator can be purchased by a hospital, and thus the ratio of simulator to learner is usually low. Alternatives include self-built models and 3D printed models, which have already been evaluated for bronchoscopy training [15, 17–19]. To ensure the step-wise structure of the workshop, two different models were used, as described in the following sections.

### Wooden box

**Fig. 1:**
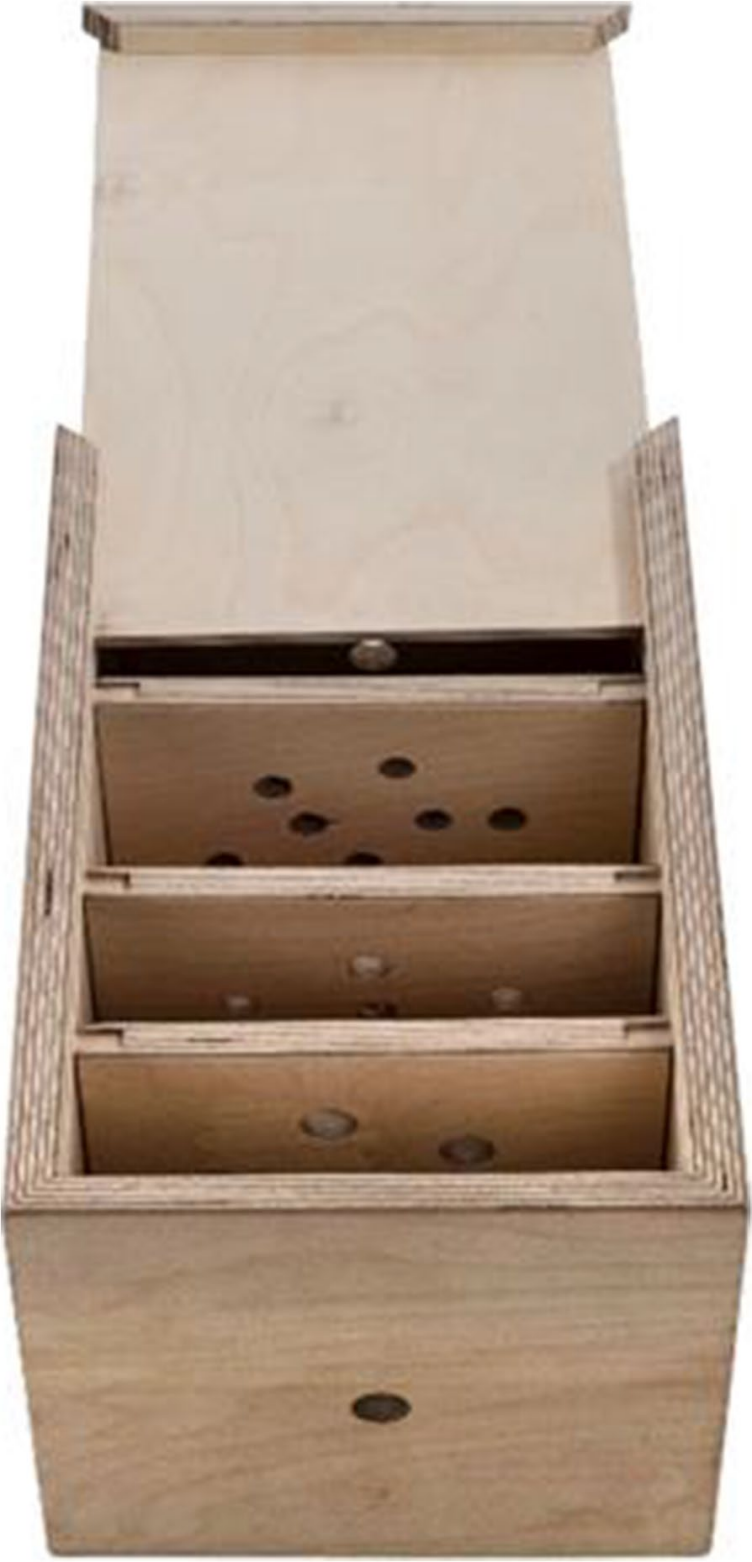
The wooden box

**Fig. 2:**
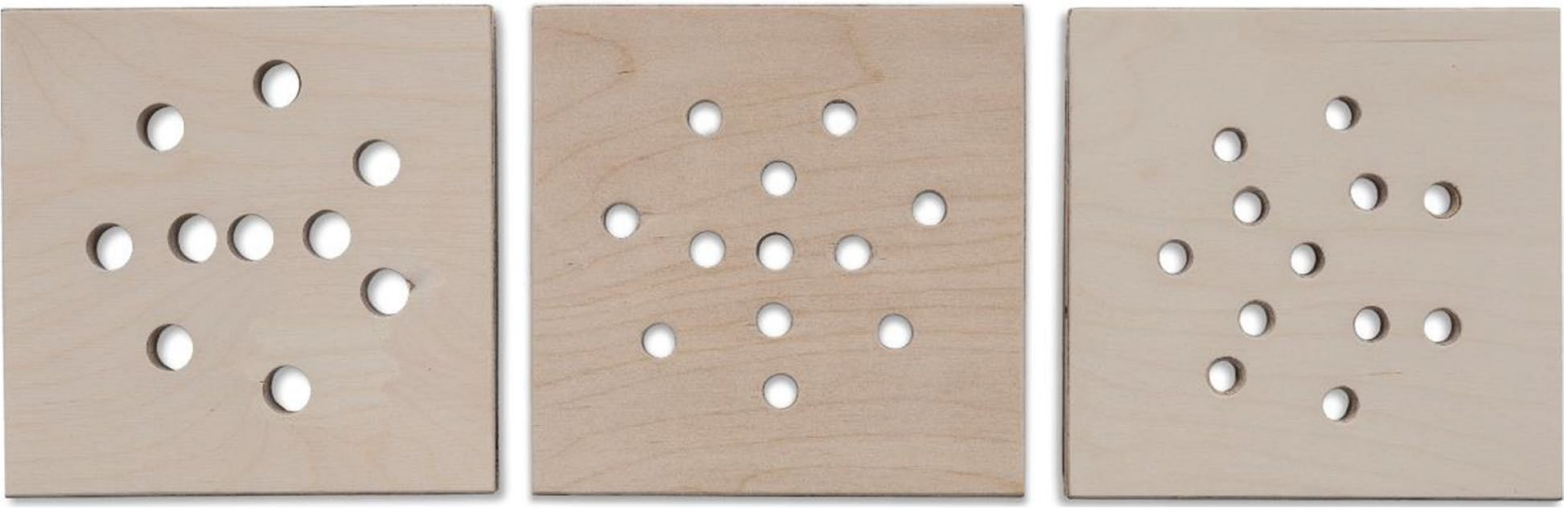
The boards of the wooden box. Holes with diameters of 18, 13 and 10 mm

Based on the model that Naik et al. [19] used, and with the help of a carpenter, one of the authors (AE) built a wooden box with a sliding lid (Fig. 1). This contained three wooden boards that could be exchanged (Fig. 2). Holes in different sizes were drilled into the front part of the box and into the three boards, to resemble the conical shape of the bronchial tree. During the initial practical part of the workshop, the residents used the box with the lid either opened or closed, to allow or block the visual control of the bronchoscope movements.

### Three-dimensional model

**Fig. 3:**
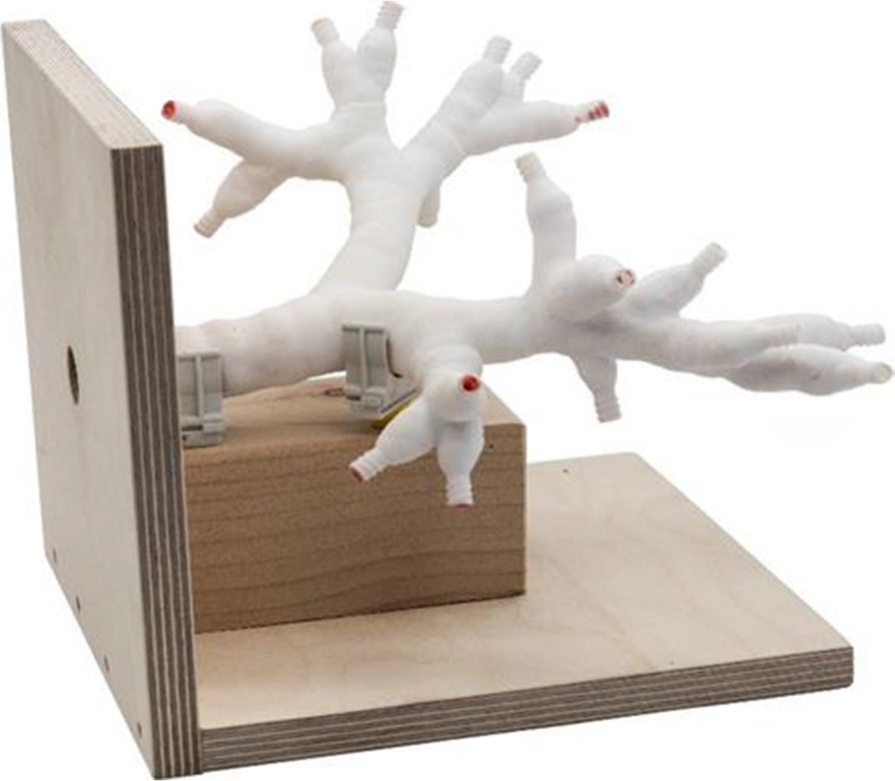
The three-dimensional printed airway

Since 3D printing was introduced, it is possible to set up a 3D airway model for a fraction of the price of a manikin [15, 18, 20]. Moreover, Osswald et al. printed their 3D airway model to be as close to reality as possible, although still at low cost [13]. For the present study, one of the authors contacted the authors of the study of Osswald et al., whereby a vector file of a 3D human airway was provided for use in the present study. The printing was carried out by Shapeways™ (Eindhoven, NL) using durable nylon plastic (PA 2200; €99 per model; Fig. 3). The inside of this model was coated with silicone rubber and was painted red, following the instructions of Osswald et al. [13].

### Data collection

As there were only a limited number of residents with little or no bronchoscopy experience within our hospital, a qualitative approach appeared to be particularly suitable. In this setting, using focus groups as the research technique allowed for, ʻexplicit use of group interactions to produce data and insights that would be less accessible without the interactions found in a groupʼ [21].

Therefore, three focus groups were held in February 2020, which lasted 2 h each. Beforehand, the residents were informed about the background and aim of the investigation. It was highlighted that participation was voluntary and would not influence their post-graduate training. All of these residents gave written consent to participate. The moderator (AE) asked the participants to take notes before answering a question, to reduce peer pressure. Moreover, the moderator encouraged all of the residents to take part, and emphasised that all opinions – including critical ones – were valued. To ensure consistency between the focus groups, the questions were asked according to a question route [22] that was also given to the residents, and that contained the main questions as shown in Table 2. All of the focus group sessions were video- and audio-recorded; in addition, the moderator (AE) took notes.

**Table 2:** Question route used for focus groups

How much experience with bronchoscopy did you have before the workshop? How did you experience the bronchoscopy workshop?	
The workshop had four phases:	
- Presentation of the bronchoscope (set-up, preparation for bronchoscopy)	
- Exercises using the wooden box	
- Introduction to the anatomy of the bronchial tree	
- Practice on the 3D printed model of a bronchial tree	
Which factors helped in your learning during these phases? Were there any factors that tended to hinder your learning?	
Which aspects of the workshop were helpful for your learning? How did you experience the sequence?	
What did you expect to learn from the workshop? What did you learn from the workshop?	
Did you enjoy the workshop? For what reasons would you recommend the workshop to other residents in our clinic?	
How could the workshop be further improved? In an ideal world with infinite financial and human resources, how should a bronchoscopy workshop be designed?	

### Qualitative analysis

A reflexive thematic analysis based on Braun and Clarke was used to analyse the data [23]. This is theoretically flexible, and can be adapted to different needs [24]. In the present study, a combined deductive–inductive semantic approach was adopted to use the intentionally provided content of the data, and at the same time, to use already existing frameworks. In this case, the analysis was carried out from the perspective of CLT, because the workshop was planned with the help of this framework.

The study assistant first created a verbatim transcription of all of the recordings. The transcripts were then coded using MAXQDA 2020 (version 20.2.2, VERBI Software, Berlin, Germany). The thematic analysis was performed following the six steps described by Braun and Clark [23]. In the first step AE and NW read the transcript several times. During step two, initial codes were generated. In the third step, codes with a relation to one another were collated and initial themes generated. In steps four and five, all of the themes were revised and refined. This process was iterative until consensus regarding the themes and subthemes was reached between the authors. In step six, AE wrote the first draft of the manuscript.

### Researchers

As the researchers had active roles in the qualitative data collection and analysis, it is important to also provide their information. The study group comprised three researchers, two of whom (AE, SH) are physicians. AE is a specialist in anaesthesiology and was the instructor of the workshops and moderator of the focus groups. AE is a consultant in the Department where 11 of the 12 residents were being trained as anaesthetists. However, AE did not have personnel responsibility for the residents. The second researcher (NJW) is a research intern who studies psychology. The last author (SH) is a paediatrician who has wide experience in medical education, including qualitative research.

## Results

A total of 12 residents took part in the half-day workshop and the focus groups. Five of the residents were male, seven were female, with ages from 24 years to 43 years. Most of these residents had no bronchoscopy experience, although some had undergone a little exposure, in terms of confirmation of correct placement of a double-lumen tube using a bronchoscope.

**Fig. 4:**
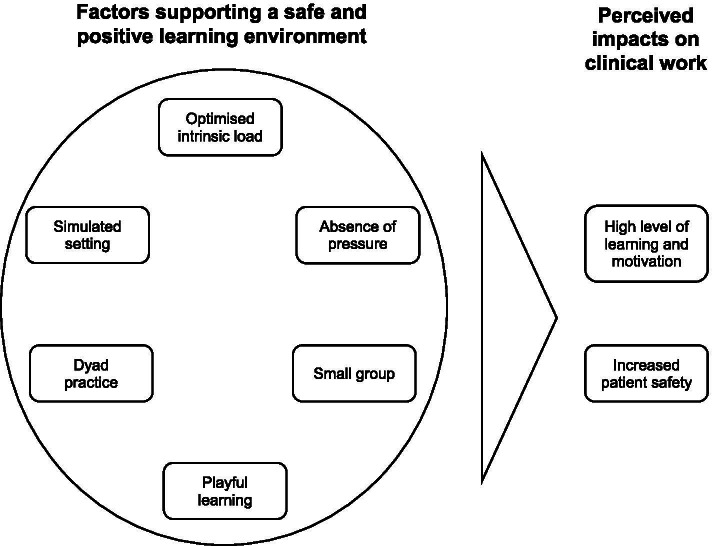
Visualisation of the results of the study. Bold font: themes; normal font: subthemes

Analysis of the data for the factors that supported learning, outcomes and acceptance resulted in two themes: (1) the factors that support a safe and positive learning environment, which included six subthemes; and (2) the perceived impacts on clinical work, which contained two subthemes. Visualisation of these aspects is shown in Fig. 4. The residents perceived no factors that hindered their learning during the workshop.

For each section quotes from the participants are provided anonymised to ensure confidentiality. Letters and numbers at the end of each quote indicate which focus group session (FG) and which participant (P) has been cited.

### Theme 1: factors supporting a safe and positive learning environment

A safe and positive learning environment encourages and supports the learner, allows learning through trial and error, and prevents negative influences. In our setting, the creation of such a learning environment was based on several factors, which are described in the following:

#### Optimised intrinsic load

The structure of the workshop with its four parts, the alternation of theory and practice, the step-wise approach, and the increasing complexity helped the residents in their learning.

“And this, where I am at the moment, does not play a role in this box. What matters is that you focus on the first two points, namely how to deal with the material and how to apply it.” (FG 3, P12)

Also, with the theory required for the practical parts presented in direct temporal connection with the practice, this contributed positively to the learning process.

“And I found it very, very helpful to be shown for the first time how many openings [segmental bronchi] I have to see [...]. And then to actually practice this on a real human model.” (FG1, P2)

#### The simulated setting

The participants described various aspects of the conducting of the workshop in a simulated setting as reasons for “stress-free learning”. When learning on a real patient, both the environment and the patient were perceived as stressors. In particular, noises such as alarm sounds, distraction by colleagues, and coughing of the patient were mentioned here. The participants did not experience these factors during the workshop, which they viewed as positive.

“One is much more focused on what we are supposed to do and not on the thousand things around it. It is that you really only have to concentrate on that and it is okay that you only focus on that and do not ignore everything else around it.” (FG2, P6)

The possibility of visual (self-)control was also expressed as beneficial. The bronchoscope position could be checked with the wooden box with the lid open, and in the 3D model it could be checked via diaphanoscopy. This was used to familiarise them with the range of movement of the bronchoscope, and as a control to see whether the goals set were reached.

Two other factors that the residents highlighted were the possibility of repeating sequences many times, and the lack of concern about injuring a patient.

“In contrast to the realistic patient, you can really go through everything. [...] Here you could go in, out, in, out. Explore all segments to your heart's content. And you do not have that under real conditions.” (FG3, P10)

### Absence of different types of pressure

In all of the focus groups, a central issue was the absence of pressure during the workshop.

“Because there were only two of us at the model and not ten people were standing around, in the second line, waiting for this model. There was no time pressure and no feeling that people watched and perhaps evaluated what you were doing.” (FG2, P5).

The residents indicated that they experienced different types of pressure when learning directly on real patients. These were indicated as mainly time pressure, the pressure to succeed, and pressure of not meeting expectations. During the workshop, the residents felt that these types of pressure were no longer present.

“That you also had that explained to you in peace and quiet, because in everyday hospital life, either it's a stressful situation, an emergency situation, but also not... You don't always necessarily have time, either.” (FG2, P7)

#### Dyad practice

Working in pairs (i.e., dyad practice) offered the possibility of mutual correction, jointly solving problems, and sharing success.

“And you can always ask your colleague: do you have an idea or suggestion on how it can be developed further or what we can do next?” (FG1, P4)

It was also noted that the learning did not only take place in the phases where they were acting as the bronchoscopist. By observing the learning partner, it was possible for the observer to then adopt successful strategies and avoid less successful ones.

“I actually found it quite exciting to see it from the other perspective. To see from the non-bronchoscopist, so to speak, where the errors of thought were, or... And I was second in line, and then I had this... okay, I need not only to look where I want to go but rather stay straight and look with the entire bronchoscope.” (FG2, P6)

#### Small group setting

The residents described the group size as ideal.

“That would be chaotic, because from such groups of ten people, there is always at least one who is more experienced, who knows everything and interferes. That's always the case. Small groups are better.” (FG1, P4)

In their opinion, an expansion by two to a total of six participants would also have been possible. It was positively perceived that the use of two bronchoscopes meant that the time per participant for using a bronchoscope was high.

Furthermore, it was appreciated that due to the small number of people in the room, there was little restlessness and distraction, and that the instructor was available at short notice.

“You simply have less time to practice [in a bigger group]. [...] Actually, it is also good when I do a bronchoscopy, she watches and tells me, there you are and if we have a question somehow, then you [AE] are asked.” (FG1, P1)

The similar learning level of the residents, and thereby their reduced fear of embarrassing themselves in front of the other participants, was also experienced as beneficial.

#### Playful learning

The participants used the wooden box in particular in a playful way, by assigning tasks to each other. The observer marked a hole with a finger, which the bronchoscopist had to find and pass through, and was thereby navigated through the box.

“That was also my thought that we set ourselves tasks, that was somehow the strength and the beauty of it.” (FG3, P10)

Another well-appreciated aspect was the possibility to test the limits of the range of movement of the bronchoscope.

“What I really liked about the box was that you could try out the extremes. You could test yourself; you could twist [the bronchoscope] into all possible positions, turn it around, and see if you could try the outermost holes.” (FG3, P9)

### Theme 2: perceived impacts on clinical work

As a result of this safe and positive learning environment that was provided, the residents felt better prepared to continue their learning of bronchoscopy on a real patient, with, in their opinion, reduced risk of harming the patient at the same time.

#### High level of learning and motivation for further learning on real patients

Some of the residents reported that through the workshop they had understood the systematics of bronchoscopy, and had started to develop their own structure for the examination of the airway while practising on the 3D model. Many were also excited about the progress they had already made in this short period of time.

“That is true, and I was also surprised that it worked out so well in the end. If you (P7) said, “go there” then, that one got there. Before, I did not think that this would work.” (FG2, P8)

The residents also mentioned that they felt motivated to continue to improve their skills on a real patient.

“It is different with the patient because you can also manage the function [of the bronchoscope], [...] rinsing, sucking, [...] that's practically the next thing you can do and if that could be done in at shorthand notice [...], that would be quite appropriate.” (FG3, P9)

Regardless of their previous experience, all of the residents said that they had learned a lot.

#### Increased patient safety

All of the participant groups emphasised that learning in a workshop, as opposed to the traditional apprenticeship model, contributes to increasing patient safety. After practising handling the bronchoscope, they thought that it was less likely that they would cause complications in a real patient.

“Because, if I have no idea what I want to see in it or where I am going or what I am doing or if I do not know how to handle it, then I endanger or injure the patient.” (FG1, P2)

Also, it was important for them that the same bronchoscopes were used in the everyday life in the hospital.

“Knowing the device means being able to see potential sources of error during preparation. [...] So the better you know the material, the better prepared you are, and there will be fewer, I would say, mistakes.” (FG3, P12)

#### Further findings

When asked for the ideal design of the workshop, the residents suggested only modifications to the wooden box. For example, the holes in the wooden boards can be marked in colours, thus providing routes of different degrees of difficulty, similar to the grading colours of ski slopes.

When asked whether they would recommend the workshop to other residents, all of the participants answered in the affirmative. The training took away their inhibitions and fear, and gave them confidence in handling the bronchoscope. They also enjoyed learning this way.

## Discussion

Complex psycho-motor skills can be very demanding to learn for novices, and can lead to cognitive overload [1]. This project aimed to reduce the likelihood of cognitive overload through an investigation of how residents perceive learning bronchoscopy in a step-wise simulated approach. The factors that were perceived as supporting a safe and positive learning environment were: optimised intrinsic load, simulated setting, absence of pressure, dyad practice, small groups, and playful learning. The perceived impacts on their clinical work were the high level of learning and increased patient safety.

Learning complex motor skills like bronchoscopy in the traditional apprenticeship model has several challenges: (1) A high intrinsic or extraneous load, or a combination of both, during the learning process can lead to cognitive overload. In the worst case scenario, this can result in the Supervisor needing to taking over, while the resident learns little and might become demotivated. (2) In everyday clinical life, the time available for training decreases as the workload increases steadily. Therefore, there is a need to set up feasible training that enhances learning, while reducing the time and personnel requirements. (3) Allowing novices to learn directly on patients comes with the risk of patient injury, and it is therefore contradictory to the growing awareness of patient safety. According to the results from the present study, these challenges can also be addressed through this workshop set-up.

During the learning of complex motor skills directly on patients (e.g., central-venous line insertion), the residents in the present study perceived multiple stressors: environmental and patient factors, time pressures, pressure to succeed, and pressure of not meeting expectations. These were avoided in this simulated setting, which contributed to a safe and positive learning environment. It is well known that stress can impair learning [25, 26]. Learning bronchoscopy and similar motor skills in a simulated setting has been shown to be more productive than the traditional apprenticeship model [11, 27]. One reason for this might be the reduction in the extraneous cognitive load that is imposed on the learner in the normal clinical setting [5, 28].

The residents perceived the workshop structure as beneficial for their learning, including the step-wise increasing complexity of the tasks, the alternation between theory and practice, and their direct temporal connection. The ʻbreakdown of the skill into its component partsʼ was identified by Wiscombe et al. as one strategy that was adopted by respiratory trainees while learning bronchoscopy [29]. These results are consistent with CLT, where part-task performance, step-wise approaches and simple-to-complex sequencing are recommended to reduce the intrinsic cognitive load [4, 5, 8]. Another means to reduce the intrinsic cognitive load is to provide new knowledge directly before it is needed to carry out a task. This was done in this workshop, and it is known as ʻjust-in-time information presentationʼ [7].

Two other factors of this set-up were highly valued by the residents: the small groups, and the dyad practice. These and other factors (e.g., simulated setting, absence of pressure, playful learning) promoted a safe and positive learning environment by reducing the fear of embarrassment (see Fig. 4). This is also one of the key features of psychological safety, which was first described by Schein and Bennis in 1965 [30]. Since then, various studies have shown that psychological safety supports both individual and organisational learning, and that it has become increasingly important in medicine and medical education [31–34].

Other advantages of the small group and the dyad practice mentioned by the residents were the high amount of time per person spent handling the bronchoscope, the feedback that they gave each other, the problem-solving together, the shared success, and the learning by observing others. These findings are in line with the literature on teaching in small groups [35] and the efficiency of learning in pairs, where observation is important to the benefits of dyad practice [36–38]. In CLT, such learning by observing somebody else is known as the human movement effect [39, 40]. This effect appears to arise from the mirror neurons, which react in the same way when watching or performing a task [41].

The residents used the wooden box in particular to engage in a playful way of learning, by setting each other tasks, and testing the limits of the bronchoscope. Computer games are also known to enhance player motivation and engagement [42]. Thus, using features of games to facilitate learning has become popular in medical education in more recent years [43, 44].

As perceived by the residents, the main impacts of the safe and positive learning environment were the high level of learning and the increased patient safety. The residents described how the workshop exercises had enabled them to develop a mental scheme for examining the airway. This made them feel more secure in handling the bronchoscope. Furthermore, residents were also surprised about the progress they had already made in handling the bronchoscope. These results fit in with previously published data that have shown that different bronchoscopy training types (e.g., modelling examples, self-training, virtual reality training) lead to enhanced basic skills in handling the bronchoscope [14, 16, 45].

Furthermore, this workshop contributed to an increase in patient safety from the perspective of the residents. This is in line with the large body of literature that has described positive effects of simulation on patient safety [27, 46–50]. All of the participants here valued the workshop as important for their post-graduate training, and they indicated that they would recommend it to fellow residents.

The participants made suggestions how to modify the wooden box, but they did not mention any factors that hindered their learning. A reason for this might be that self-assessment is not always accurate as shown in several studies [51, 52].

### Strengths and limitations

This study investigated residents’ perception of their learning of basic bronchoscopy skills through this combination of teaching approaches with CLT principles. To the best of our knowledge, this has not been reported before. Furthermore, according to the residents, the three main challenges of teaching bronchoscopy were successfully addressed using this approach: managing the cognitive load; setting up feasible training; and adhering to the principles of patient safety.

However, this study also has some limitations. We used an exclusively qualitative approach to analyse and interpret the perceptions of the residents. Additional research can investigate a similar workshop setting under quantitative aspects, and thereby add another perspective. Furthermore, AE was the instructor of the workshops and moderator of the focus groups, and was at the same time a Consultant in the Department where 11 of the 12 residents were training as anaesthetists. To reduce the influence on the residents’ answers, AE explicitly indicated in the focus groups that all of the views expressed (also if critical) were equally important, and encouraged the participation of all of the residents. To avoid any influence in future studies, one person should lead the workshops and another person should moderate the focus groups. Moreover, the focus group moderator should not be in a direct superior position to the participants.

## Conclusions

We designed and set up a half-day workshop to teach the basics of bronchoscopy while avoiding cognitive overload, which often occurs when learning bronchoscopy in the traditional apprenticeship model. CLT findings were taken into account to achieve this, and both the intrinsic and extraneous loads were reduced. From the residents’ point of view, this created a safe and positive learning environment, which was characterised by optimised intrinsic load, simulated settings, absence of pressure, dyad practice, small groups, and playful learning. In addition, they perceived that this approach led to high levels of learning, plus motivation for further learning on real patients, and will have increased patient safety. Additional research can investigate whether these results can be confirmed in studies with a quantitative design.

## Data Availability

The datasets analysed during the current study are available from the corresponding author on reasonable request.
